# ABA Alleviates Uptake and Accumulation of Zinc in Grapevine (*Vitis vinifera* L.) by Inducing Expression of ZIP and Detoxification-Related Genes

**DOI:** 10.3389/fpls.2019.00872

**Published:** 2019-07-05

**Authors:** Changzheng Song, Yifan Yan, Abel Rosado, Zhenwen Zhang, Simone Diego Castellarin

**Affiliations:** ^1^Shaanxi Engineering Research Center for Viti-Viniculture, College of Enology, Northwest A&F University, Yangling, China; ^2^Wine Research Centre, The University of British Columbia, Vancouver, BC, Canada; ^3^Department of Botany, The University of British Columbia, Vancouver, BC, Canada

**Keywords:** abscisic acid, ABA mimic, excess Zn stress, grape, heavy metal toxicity

## Abstract

Abscisic acid (ABA) is a plant hormone that can mitigate heavy metal toxicity. Exogenous ABA and ABA mimic 1 (AM1) were applied to study the influence on Zn uptake and accumulation in *Vitis vinifera* L. cv. Merlot seedlings exposed to excess Zn. The seedlings were treated with either normal or excess levels of Zn in combination with applications of ABA and AM1. Excess Zn exposure resulted in decreased lateral root length, decreased photosynthesis, elevated uptake, and accumulation of Zn in roots, trunks, and stems, decreased jasmonic acid content in roots and leaves, and induced the expression of Zn transportation- and detoxification-related genes. Remarkably, in the presence of toxic amounts of Zn, the exogenous application of ABA, but not of AM1, reduced the uptake and accumulation of Zn in roots and induced higher expression of both ZIP genes and detoxification-related genes in root and leaf. These results indicate that exogenous ABA enhances the tolerance of grape seedlings to excess Zn and that AM1 is not a suitable ABA mimic compound for Zn stress alleviation in grapes.

## Introduction

Wine grapes are produced in more than 40 countries. Climates and soils of different regions contribute to the diversity of quality and type of the grape and wines (Anderson and Aryal, 2015). In the last decades, wine grape cultivation has been rapidly developed in tropical and subtropical areas, including Mexico, Venezuela, Peru, Brazil, India, Thailand, and South China (Intarapichet et al., 2007; Jogaiah et al., 2013; Terra et al., 2013; dos Santos Lima et al., 2014), driven by local wine market demand (Anderson and Aryal, 2015; Anderson and Nelgen, 2015). However, soils in these areas are often susceptible to heavy metal toxicity because of low pH and pollution.

Zinc (Zn) is an essential micronutrient for plants. It plays a key role in photosynthetic redox reactions, and it is an essential cofactor for many enzymes involved in nitrogen metabolism and protein synthesis (Hafeez et al., 2013). Similar to other plant micronutrients, Zn is beneficial in a narrow range of concentrations, and its bioavailability in soils increases at low pH (Alloway, 2012). In unpolluted soils, the amount of Zn is generally below 125 ppm (Baccio et al., 2003; Hussain et al., 2010). However, environmental pollution due to industrial and agricultural activities including excessive application of Zn-containing fertilizers and pesticides, manures, sewage sludge, smelters, incinerators, mines, and galvanized products has increased the concentration of Zn in many agricultural soils above the threshold of phytotoxicity (Robson, 1993a).

According to the no observed effect concentrations (NOECs), the highest Zn dose that can be added to soils without affecting plants is 32–400 mg/kg (fresh weight). Toxic effects are identified at total Zn concentrations of 100 to >1000 mg/kg (Alloway, 2012), and a tissue concentration ≥400 mg/kg (dry weight) of Zn is considered toxic for nearly all plants (Sofo et al., 2013). Generally, Zn phytotoxicity inhibits the growth of roots and stems, modifies leaf morphology, induces chlorosis, reduces photosynthesis, interferes with the uptake of other nutrients, induces stress phytohormones, and alters the expression of genes related to Zn accumulation and detoxification (Rout and Das, 2009).

Abscisic acid (ABA) is a plant hormone with important functions as a stress alleviator, particularly in responses to drought, salt, and chilling stress (Bari and Jones, 2009). ABA levels in plants are tightly controlled by environmental conditions, and high ABA concentration activates signaling cascades of other phytohormones, such as salicylic acid (SA) and jasmonic acid (JA) (Shi et al., 2015). Exogenous ABA application causes an alleviating effect on plants under heavy metal stresses (Hsu and Kao, 2003; Wang et al., 2013), and a recent study by Shi et al. (2015) suggests that exogenous ABA applications can decrease the phytotoxic effect of Zn in *Populus × canescens* tissues by modulating the transcriptional activity of key genes involved in Zn transport and detoxification, and by activating the antioxidative defense system.

Since the application of ABA in agricultural practice is limited by ABA’s chemical instability, costly production, and rapid catabolism, a small molecule, ABA mimic (AM1), has been recently identified as an ABA surrogate based on its structural analogy to ABA (Cao et al., 2013). Similar to ABA, AM1 activates multiple members of the ABA receptor family, such as pyrabactin resistance 1 (PYR1) and PYR1-like (PYL) protein, and enhances the tolerance of plants to drought and cold stress (Cao et al., 2013; Cheng et al., 2016). However, compared with ABA, AM1 is easier to synthesize and more resistant to photolysis. Therefore, it has the potential to become an ABA replacement in agricultural practice.

In the present study, the effects of ABA and AM1 in heavy metal stress alleviation were tested by studying the uptake and translocation of Zn in “Merlot” grapevines grown under excess Zn stress. Leaf area, photosynthesis and foliar pigments, Zn localization and concentration, phytohormone level, and expression of Zn-related genes were measured to elucidate the physiological and molecular response underlying the potential mitigating effects of ABA on Zn uptake and on physiology in grapevine, and to explore strategies to mitigate Zn phytotoxicity in vineyards.

## Materials and Methods

### Plant Cultivation and Treatments

One-year-old hardwood cuttings of “Merlot” (*V. vinifera* L.) with 4–6 nodes were pre-rooted in a thermostatically controlled heated container (26°C at the base of the cuttings) in a cold room (4°C) for 40 days. The cuttings were then transferred to pots and cultivated for 5 weeks in the Horticulture Greenhouse at The University of British Columbia (26°C day and 20°C night, 16 h photoperiod). Afterward, the rooted seedlings were transferred into 4 L plastic pots filled with clean sand and cultivated for 10 more weeks. The plants were irrigated with 50 mL distilled water or Hoagland solution alternately at each sunset. Thirty-two plants with similar heights were randomly divided into four groups and treated with either basal (0.765 μM Zn^2+^, 0.22 mg/L) or excess levels (10 mM Zn^2+^, 2880 mg/L) of zinc sulfate (ZnSO_4_.7H_2_O) dissolved in aqueous solution. To 2 of 3 excess Zn treatments, 10 μM ABA or 10 μM AM1 solutions were applied to the roots. This resulted in four treatments: Basal Zn, Excess Zn, Excess Zn + ABA, and Excess Zn + AM1. The treatments were applied in combination with the Hoagland solution every day for 10 days. Eight grapevines per treatment were considered. Four plants per treatment were harvested on the 4th day after the treatment began (DAT); the rest of the plants were harvested on the 10th DAT. Each plant was regarded as a biological replicate, so four biological replicates were included in each treatment.

### Leaf Area, Root Length, and Gas Exchange Measurement

For each plant, the lengths of the shoot and of the main vein of each leaf were measured using a ruler at 0, 4, 10 DAT. A regression line between the length of the main vein and the total leaf area was calculated by measuring 50 leaves of different sizes using a ruler and leaf area meter (LI-3100, LI-COR, NE, United States) (Sivilotti et al., 2016). The regression was used to estimate the leaf area of each leaf of the plants in a non-destructive manner, and the total leaf area per plant was calculated by summing the area of each leaf of the plant. The length of lateral roots was also measured at 10 DAT.

Before each sampling point, the leaf gas exchange was determined for each plant. Measurements were conducted from 9:00 am to 1:00 pm. Mature leaves with plastochron 7–9 (Lamoreaux et al., 1978) were selected for the measurement of the net photosynthetic rate (*A*), the stomatal conductance (*gs*), and the transpiration rate (*E*) using a Li-Cor-6400 portable photosynthesis system (Li-COR Inc., NE, United States). The light source from the lamp was set at 1500 μmol/m^2^/s, air flow through the sample chamber was 500 μmol/s, reference cell CO_2_ concentration was 400 μmol/mol, and the leaf temperature was 22.0°C.

### Sampling of Plant Material

Lateral roots, trunks, and stems between the 5th and 9th nodes from the shoot tip, and leaves between the 1st and 10th node, were harvested at 4 and 10 DAT. Subsamples of fine fresh root, trunk, stem, and leaf were harvested for histochemical analysis. The rest of the samples were frozen in liquid nitrogen and ground into powder using RNase-free mortars and pestles, and then stored at -80°C. This plant material was used for Zn and hormone determination and gene expression analysis. Additional leaves with plastochron 7–9 were collected and used for pigment determination.

### Analysis of Foliar Pigment

The leaves were cleaned with distilled water after collection. The veins were removed, and an aliquot of 0.5 g of leaf sample was added into a mortar and ground with 80% acetone. The homogenate was washed out with 80% acetone, filtered into a 10 mL volumetric flask, and then filled to volume. The concentrations of chlorophylls were determined spectrophotometrically as previously described (Wellburn, 1994).

### Determination of Zn Concentration

Powder samples, approximately 5 g each, were dried at 60°C for 72 h to calculate the fresh to dry mass ratio. Afterward, 1 g of dried sample was weighed into a tared, oven-dried crucible. The samples were ashed in the muffle furnace for 1 h at 300°C and 4 h at 500°C. After the samples were cooled in a desiccator, 5 mL of 2 M HCl was carefully added. A warm sand bath was used to dissolve soluble constituents, and a tared Waterman #42 paper was used to filter the solution into a 100 mL volumetric flask. Distilled water was used to wash the filter paper and make up to 100 mL of solution. Subsequently, the concentrations of Zn and other mineral elements were determined using a Nu AttoM Inductively Coupled Plasma Mass Spectrometer (ICP-MS) (CAMECA, Gennevilliers, France) as described by Murray et al. (2000). For Zn levels in leaf, the concentrations were measured in the petiole, which has been widely used for diagnosis of Zn in previous research (Alloway, 2004; Romero et al., 2013).

### Analysis of Zn Localization

Zn accumulation was histochemically detected in root, trunk, stem, and leaf tissues using the Zn chelating agent dithizone (diphenylthiocarbazone, 30 mg dissolved in 60 ml acetone, 20 ml distilled water and four drops of glacial acetic acid). Hand sections of fresh samples were stained for 2 min and rinsed several times with deionized H_2_O. Afterward, the sections were immediately analyzed with an Olympus AX-70 light microscope (Olympus Corporation, Tokyo, Japan). The red–purple Zn dithizonate complex was photographed under the light microscope at 10× or 20× magnification using an Olympus Fluoview 1000 scan head connected to a computer (Olympus Corporation, Tokyo, Japan).

### Determination of Phytohormone Contents

#### Chemical Standards

ABA (cat. no. 013 2701) was purchased from OlChemIm Ltd. (Olomouc, Czechia), SA (cat. no. S5922) from Sigma-Aldrich (ON, Canada), and JA (cat. no. 88300) from Cayman Chemical (MI, United States). d_6_-ABA (cat. no. A110002) was purchased from Toronto Research Chemicals (ON, Canada), d_4_-SA (cat. no. CS-O-06948) from Clearsynth (ON, Canada), and d_5_-JA (cat. no. D-6936) from C/D/N Isotopes Inc. (QC, Canada).

#### Phytohormone Extraction

ABA, SA, and JA were extracted according to the method published by Shi et al. (2015) with minor modifications. An aliquot of 0.4 g of fresh tissue was extracted by 5 mL of 80% (v/v with water) methanol containing 200 mg/L of butylated hydroxytoluene and 500 mg/L of citric acid monohydrate. Aliquots of 100 ng of d_6_-ABA, d_4_-SA, and d_5_-JA each were added to the extraction buffer as internal standards (IS). The samples were shaken for 24 h at 4°C and subsequently centrifuged for 15 min at 10,000 × *g* under 4°C. The supernatant was collected and transferred into a 20 mL flat-bottom vial using a syringe (Luer-Lok Tip Syringe, Sigma-Aldrich, ON, Canada) and filter (0.22 μm × 13 mm, PVDF Millex Filter, Sigma-Aldrich, ON, Canada). Afterward, the supernatant was concentrated by a Thermo Savant’s Universal Vacuum System (UVS400) (Thermo Fisher Scientific, Waltham, MA, United States) and re-suspended in 250 μL of 80% methanol.

#### Identification and Quantification of Phytohormones

For the identification and quantification of ABA, SA, JA, and AM1, 5 μL of extract was injected into an Agilent 1100 Series high performance liquid chromatograph (HPLC) coupled to an LC/MSD Trap XCT Plus mass selective detector. Chromatography separation was carried out by an Agilent ZORBAX SB-C18 Column (1.8 μm, 4.6 mm × 50 mm). Mass spectrometry data were generated via electrospray ionization (ESI) in negative modes. The temperature of the column was maintained at 60°C. Mobile phases consisted of Solvent A and B. Solvent A was distilled water with 0.2% formic acid; solvent B was acetonitrile with 0.2% formic acid. The LC separation used a binary solvent gradient with a flow rate of 1.00 mL/min. The gradient conditions were 1.00 min, 10.0% solvent B; 5.00 min, 90.0% solvent B; 6.00 min, 90.0% solvent B; 6.10 min; 5.0% solvent B. Extracted ion chromatography was used for ABA, SA, and JA quantification. Specifically, [MS-H^+^] 263 was used for ABA, [MS-H^+^] 269 was used for d_6_-ABA, [MS-H^+^] 137 was used for SA, [MS-H^+^] 141 was used for d_4_-SA, [MS-H^+^] 209 was used for JA, [MS-H^+^] 214 was used for d_5_-JA. Hormone concentrations were calculated based on internal standard-based calibration curves (Supplementary Table S1) that were prepared for each analyte/IS pair, as described elsewhere (Ross et al., 2004). Samples were run in random order.

### RNA Extraction and Analysis of Transcriptional Level by Real-Time PCR

Total RNA extraction and quantitative real-time PCR (qRT-PCR) were performed as described by Savoi et al. (2016). Total RNA was extracted with the “Spectrum Plant total RNA” kit (Sigma-Aldrich, ON, Canada) from 0.1 g of sample powder. The quantity and quality of the RNA were analyzed with an ND-1000 Spectrophotometer NanoDrop (Thermo Fisher Scientific, Wilmington, DE, United States). All RNA samples were digested to remove genomic DNA and reverse-transcribed in a 20 mL reaction mixture for cDNA synthesis using a DNase I, RNase-free, and RevertAid First Strand cDNA Synthesis Kit (Thermo Fisher Scientific, MA, United States), respectively, following the manufacturer’s manuals. The expression levels of 10 Zn-related genes, whose functions will be introduced in discussion, *VviZIP2, VviZIP6, VviZIP7, VviZIP13, VviHMA2, VviNAS, VviNRAMP3, VviYSL1, VviPCR2*, and *VvibZIP23*, were determined in root and leaf tissues by using an Applied Biosystems^TM^ 7500 Fast Dx Real-Time PCR Instrument (Thermo Fisher Scientific, MA, United States) and a PowerUP SYBR Green Master Mix (Thermo Fisher Scientific, TX, United States). The genes were selected according to whole-genome array data organized from publicly available RNA-sequencing experiments in our previous publication (Wong et al., 2018). The primers were designed with PrimerQuest Tool (Integrated DNA Technologies, IA, United States) (Supplementary Table S2), and the annealing temperature was 60°C for all primer pairs except *VviUbiquitin*, which annealed at 56°C.

### Statistical Analysis

Mean and standard deviation of values were calculated using Excel 2016 (Microsoft, Washington, DC, United States). One-way analysis of variance (ANOVA) and two-way ANOVA were conducted using SPSS Statistics 20.0 (IBM, NY, United States). For experimental variables, the principal factors in two-way ANOVA were treatment and time of sampling. Statistical significance among averages was evaluated using the LSD’s test with *P* < 0.05. Figures were made using the drawing software Origin 9.0 (OriginLab, MA, United States).

## Results

### Leaf Area Index and Root Length

Shoot length and leaf area were measured at 0, 4, and 10 DAT. No difference of shoot length or leaf area among treatments was observed (Table 1). However, the Excess Zn and Excess Zn + ABA treatments promoted the increase (from 0 DAT to 4 and 10 DAT) of leaf area. As for the roots at 10 DAT, the average length in all the excess Zn treatments decreased compared with the Basal Zn treatment, and no alleviating effect was found for the ABA or AM1 addition treatments.

**Table 1:** Shoot length, shoot length increment from 0 days after the treatment (DAT), leaf area, leaf area increment, and lateral root length (cm) of “Merlot” (*Vitis vinifera* L.) seedlings exposed to basal and excess Zn in combination with ABA and AM1 (*n* = 4).

Treatment	DAT	Shoot length (cm)	Shoot length increment (cm)	Leaf area (cm^2^)	Leaf area increment (cm^2^)	Lateral root length (cm)^#^
Basal Zn	4	116.38±24.78	10.63±3.46	1306.73±59.02	102.38±31.06	–
Excess Zn	4	93.71±25.90	13.29±3.77	1367.28±178.10	163.35±19.29	–
Excess Zn + ABA	4	107.67±26.30	15.33±1.63	1601.07±95.96	138.03±13.04	–
Excess Zn + AM1	4	123.38±35.13	10.88±5.87	1447.93±333.87	108.69±22.49	–
Basal Zn	10	116.75±5.68	26.00±15.19	1370.11±29.19	165.75±55.41	39.62±2.36b
Excess Zn	10	130.00±11.14	32.33±3.79	1488.85±170.36	284.92±14.54	21.94±3.04a
Excess Zn + ABA	10	145.50±24.75	28.50±2.12	1701.95±78.89	238.91±30.11	20.97±3.80a
Excess Zn + AM1	10	133.75±30.73	16.50±9.71	1530.53±326.26	191.29±43.55	19.73±2.81a
Two-way ANOVA						
*P*-value Tr		ns	^∗^	ns	^∗∗^	–
*P*-value Ti		^∗^	^∗∗∗∗^	ns	^∗∗∗∗^	–
*P*-value TrxTi		ns	ns	ns	ns	–
LSD test						
Basal Zn		–	ab	–	c	
Excess Zn		–	a	–	a	
Excess Zn + ABA		–	a	–	ab	
Excess Zn + AM1		–	b	–	bc	

*Two-way ANOVA was performed and the *P*-values of the effects of Zn treatments (Tr), time of sampling (Ti), and their interaction (TrxTi) are indicated: ^∗^*P* < 0.05; ^∗∗^*P* < 0.01; ^∗∗∗^*P* < 0.001; ^∗∗∗∗^*P* < 0.0001; ns, *P* > 0.05. Different letters indicate different averages based on LSD *post hoc* tests. ^#^A one-way ANOVA and a LSD *post hoc* test was run to test the effect of Zn treatments on lateral shoot length at 10 DAT.*

### Photosynthesis and Foliar Pigments

Leaf gas exchanges were affected by Zn treatments, but a significant Zn treatment × time of sampling interaction was also observed (Table 2). The CO_2_ assimilation rate (*A*) in the three excess Zn treatments was lower than that in the Basal Zn treatment at both 4 and 10 DAT. As for the stomatal conductance (*gs*) and the transpiration rate (*E*), the Excess Zn and Excess Zn + ABA treatments had higher *gs* than Basal Zn at 10 DAT, and the Excess Zn treatment had higher *E* than the Basal Zn treatment at 10 DAT. The foliar pigments were not affected by treatments.

**Table 2:** Leaf photosynthesis (*A*), stomatal conductance (*gs*), transpiration rate (*E*) and photosynthetic pigments at 4 or 10 days after treatment (DAT) (*n* = 4).

Treatment	DAT	*A* (μmol CO_2_ m^-2^ s^-1^)	gs (mol H_2_O m^-2^ s^-1^)	*E* (mmol H_2_O m^-2^ s^-1^)	*Chl a* (mg g^-1^ FW)	*Chl b* (mg g^-1^ FW)	*Chl (a+b)* (mg g^-1^ FW)
Basal Zn	4	11.38±0.35a	0.06±0.021b	1.99±0.57bc	1.08±0.09	0.31±0.06	0.21±0.01
Excess Zn	4	8.80±0.25b	0.06±0.005b	1.79±0.18c	1.34±0.01	0.37±0.08	0.26±0.04
Excess Zn + ABA	4	7.98±1.25bc	0.05±0.002bc	1.65±0.06cd	0.79±0.11	0.20±0.06	0.18±0.04
Excess Zn + AM1	4	4.23±0.55f	0.04±0.001bc	1.47±0.03cd	0.90±0.16	0.27±0.10	0.20±0.01
Basal Zn	10	7.28±0.36c	0.06±0.001b	1.93±0.01bc	1.31±0.10	0.35±0.01	0.28±0.04
Excess Zn	10	6.19±0.65d	0.11±0.038a	2.97±0.94a	1.12±0.13	0.31±0.08	0.24±0.05
Excess Zn + ABA	10	5.21±0.37e	0.09±0.035a	2.67±0.89ab	1.05±0.10	0.31±0.03	0.24±0.03
Excess Zn + AM1	10	5.91±0.36de	0.03±0.004c	1.04±0.15d	1.10±0.16	0.34±0.05	0.24±0.02
Two-way ANOVA							
*P*-value Tr		^∗∗^	^∗∗^	^∗∗^	ns	ns	ns
*P*-value Ti		^∗∗∗∗^	ns	^∗∗∗^	^∗^	ns	^∗^
*P*-value TrxTi		^∗∗∗^	^∗∗^	^∗^	ns	ns	ns

*Two ways ANOVA was performed and the *P*-values of the effects of Zn treatments (Tr), time of sampling (Ti), and their interaction (TrxTi) are indicated: ^∗^*P* < 0.05; ^∗∗^*P* < 0.01; ^∗∗∗^*P* < 0.001; ^∗∗∗∗^*P* < 0.0001; ns, *P* > 0.05. Different letters indicate different averages based on LSD *post hoc* test.*

### Zn Concentrations and Localization

**FIGURE 1:**
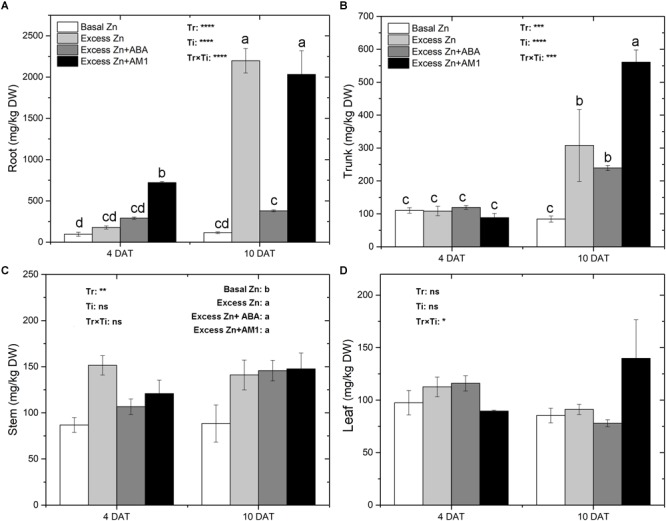
Zn concentrations (mg/kg DW) in root **(A)**, trunk **(B)**, stem **(C)**, and leaf **(D)** tissues of “Merlot” (*Vitis vinifera* L.) seedlings exposed to Zn treatments at 4 or 10 days after treatment (DAT) (*n* = 4). Two-way ANOVA was performed and the *P*-values of the effects of Zn treatments (Tr), time of sampling (Ti), and their interaction (TrxTi) are indicated: ^∗^*P* < 0.05; ^∗∗^*P* < 0.01; ^∗∗∗^*P* < 0.001; ^∗∗∗∗^*P* < 0.0001; ns, *P* > 0.05. Different letters indicate significantly different averages based on LSD *post hoc* tests.

Zn concentrations in tissues of “Merlot” seedlings were measured at 4 and 10 DAT (Figure 1). Zn treatments affected Zn concentrations in roots, trunks, and stems, but not in leaves. Major effects were observed at 10 DAT. Overall, excess Zn applications increased the Zn concentration in root, trunk, and stem tissues. In the roots, excess Zn exposure led to an increase of Zn accumulation at 4 DAT, and AM1 addition further promoted Zn uptake. At 10 DAT, the Excess Zn and Excess Zn + AM1 treatments had a sharp increase of Zn concentration; the increase was much less in the Excess Zn + ABA treatment, indicating an alleviating effect of ABA on excess Zn accumulation. In the trunk, no effects were observed in the excess Zn treatments at 4 DAT, but increases of Zn levels were observed in the excess Zn treatments at 10 DAT with no mitigating effect of ABA and with a promoting effect of AM1. In the stems, all the excess Zn treatments increased the Zn concentration, and no effect of the time of sampling or of the interaction between the treatments and the time of sampling was observed.

Zn-dithizone complexes were observed at 10 DAT in seedling tissues. As shown in Supplementary Figure S1, more Zn accumulated in epidermal cells in roots, pith rays, and phloem in trunks, stems, and petioles in the excess Zn treatments than in the Basal Zn treatment. According to the intensity of red–purple Zn-dithizone precipitates in the tissues, a sharp decline of Zn concentration was observed from lower (roots) to higher (petioles) tissues of the seedlings. Zn accumulation exhibited a similar pattern in the Excess Zn, Excess Zn + ABA, and Excess Zn + AM1 treatments.

### Phytohormone and AM1 Concentrations

**FIGURE 2:**
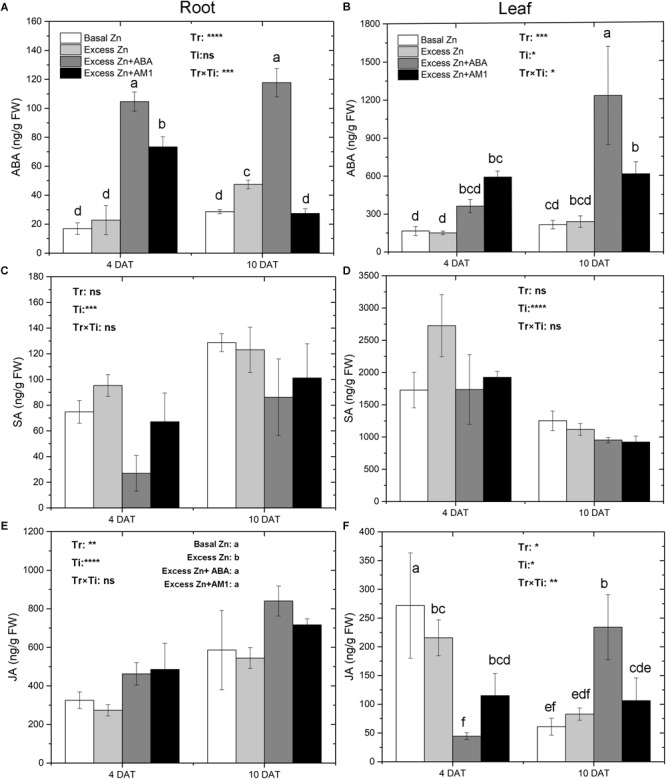
Abscisic acid (ABA), salicylic acid (SA) and jasmonic acid (JA) in the root **(A,C,E)** and leaf **(B,D,F)** tissues of “Merlot” (*Vitis vinifera* L.) seedlings exposed to Zn treatments at 4 or 10 days after treatment (DAT) (*n* = 4). Two-way ANOVA was performed and the *P*-values of the effects of Zn treatments (Tr), time of sampling (Ti), and their interaction (TrxTi) are indicated: ^∗^*P* < 0.05; ^∗∗^*P* < 0.01; ^∗∗∗^*P* < 0.001; ^∗∗∗∗^*P* < 0.0001; ns, *P* > 0.05. Different letters indicate significantly different averages based on LSD *post hoc* tests.

Excess Zn exposure without ABA addition did not lead to changes of ABA in roots and leaves at 4 DAT but slightly increased ABA in roots at 10 DAT (Figure 2A,B). Excess Zn + ABA remarkably increased ABA in roots at both 4 DAT and 10 DAT, and increased ABA in leaves at 10 DAT; however, interactions between the treatments and the time of sampling were observed. Excess Zn + AM1 promoted ABA in roots at 4 DAT. No effect of excess Zn was observed on SA in roots or leaves (Figure 2C,D). Excess Zn decreased JA in the roots compared with the Basal Zn treatment (Figure 2E). The addition of exogenous ABA and AM1 alleviated the effect of excess Zn. No difference of JA in roots was found among the Excess Zn + ABA, Excess Zn + AM1, and Basal Zn treatments. In the leaves at 4 DAT, the Excess Zn treatment slightly reduced JA compared with the Basal Zn treatment, while the addition of exogenous ABA severely decreased JA compared with the Basal Zn and Excess Zn treatments (Figure 2F). At 10 DAT, Excess Zn + ABA induced the highest JA concentration among treatments.

AM1 was detected in roots of plants treated with Excess Zn + AM1, at both 4 DAT and 10 DAT (Supplementary Figure S2), and the concentration at 10 DAT (283.33 μg/g FW) was higher than that at 4 DAT (63.89 μg/g FW). No AM1 was detected in leaf samples at 4 or 10 DAT.

### Expression of Genes Involved in Zn Uptake and Translocation

To study the effect of excess Zn and ABA or AM1 addition on the expression of Zn uptake- and translocation-related genes, 10 genes, including the members of the ZIP family of transporters *VviZIP2, VviZIP6, VviZIP7*, and *VviZIP13*, heavy metal detoxification-related genes *VviHMA2* (Heavy Metal ATPases of the P1B-type ATPases), *VviNAS* (NA Synthase), *VviNRAMP3* (Natural Resistance-Associated Macrophage Protein), *VviYSL1* (Yellow Stripe-Like), and *VviPCR2* (Plant Cadmium Resistance), as well as the basic-region leucine zipper (bZIP) transcription factor *VvibZIP23*, were considered. Based on whole-genome array data (Supplementary Table S3 and Figure S3), relative expression of a total of 68 grapevine homologs of the above genes was analyzed across tissues and experimental conditions, and the 10 selected genes were the most expressed among the homologs.

**FIGURE 3:**
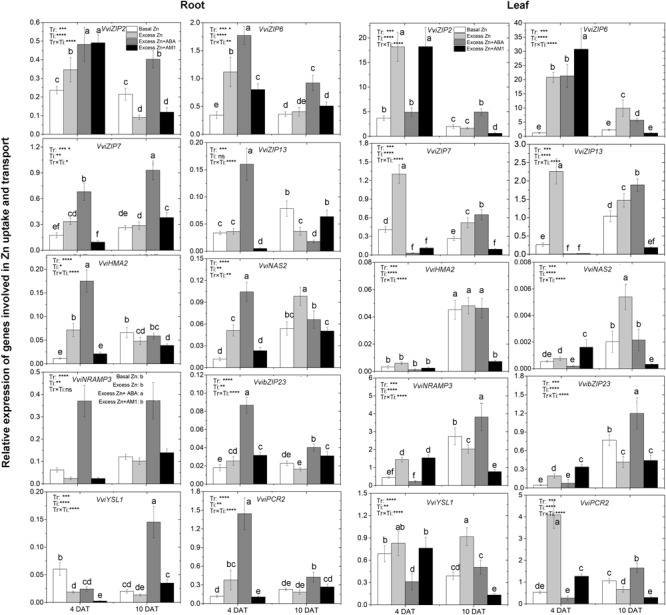
Relative expression of genes involved in Zn uptake and transport in the root (left panels) and leaf (right panels) tissues of “Merlot” (*Vitis vinifera* L.) seedlings exposed to Zn treatments at 4 or 10 days after treatment (DAT) (*n* = 4). Two-way ANOVA was performed and the *P*-values of the effects of Zn treatments (Tr), time of sampling (Ti), and their interaction (TrxTi) are indicated: ^∗^*P* < 0.05; ^∗∗^*P* < 0.01; ^∗∗∗^*P* < 0.001; ^∗∗∗∗^*P* < 0.0001; ns, *P* > 0.05. Different letters indicate significantly different averages based on LSD *post hoc* tests.

The expression of most genes was notably affected by excess Zn exposure, ABA or AM1 addition, and the sampling time in both roots and leaves (Figure 3). In the roots, excess Zn induced higher expression of all the ZIP genes but *VviZIP13* at 4 DAT, as well as some of the heavy metal detoxification-related genes, such as *VviHMA2, VviNAS, VvibZIP23*, and *VviPCR2*. *VviHMA2* and *VviNAS* were the most affected by the Excess Zn treatment – seven and five times higher than Basal Zn treatment, respectively. At 10 DAT, most of the above genes were similarly expressed in both the Basal Zn and Excess Zn treatments, but *VviZIP2, VviZIP13*, and *VviHMA2* were lower in expression in the Excess Zn than in the Basal Zn treatment. Excess Zn + ABA generally induced the expression of ZIP genes and detoxification-related genes than in Basal Zn and Excess Zn at 4 DAT. The detoxification genes *VviHMA2, VviNAS*, and *VviPCR2* were the most influenced – 10 times more expressed in Excess Zn + ABA than in Basal Zn treatment. As compared with the Excess Zn treatment, the ABA addition led to a long-lasting induced expression of these genes at 10 DAT. The Zn detoxification gene *VviYSL1* was the most induced by Excess Zn + ABA at 10 DAT. Expression of other genes, *VviZIP6, VviZIP7, VvibZIP23, VviNRAMP3*, and *VviPCR2* was also upregulated in the Excess Zn + ABA treatment as compared with the Basal Zn and Excess Zn treatments. Excess Zn + AM1 affected the expression of *VviZIP2, VviZIP6, VviZIP13, VvibZIP23, VviYSL1*, and *VviPCR2* at 4 DAT when compared with Basal Zn. The effect of AM1 addition on the expression of most genes was rarely consistent with the effect of ABA addition.

In the leaves, elevated expression of all the ZIP genes, *VviNRAM3*, and *VviPCR2* was found in the Excess Zn treatment at 4 DAT. The Excess Zn + ABA treatment alleviated the increase of expression of *VviZIP2, VviZIP7, VViZIP13, VviNRAM3, VvibZIP23*, and *VviPCR2* observed in Excess Zn at 4 DAT. Moreover, Excess Zn + ABA decreased *VviZIP7, VviZIP13*, and *VviYSL1* expression as compared with the Basal Zn treatment. In the Excess Zn + AM1 treatment, the expression of *VviZIP2, VviZIP6, VviNRAM3, VvibZIP23*, and *VviPCR2* was similar to that in the Excess Zn treatment and higher than that in the Basal Zn treatment at 4 DAT. At 10 DAT, in the Excess Zn treatment, the expression of *VviZIP6, VviZIP7, VviZIP13, VviNAS2*, and *VviYSL1* was higher than in the Basal Zn treatment, while the expression of *VviNRAMP3, VvibZIP23*, and *VviPCR2* was lower than Basal Zn treatment. The expression of most ZIP genes (*VviZIP2, VviZIP7, VviZIP13*), *VviNRAMP3, VvibZIP23*, and *VviPCR2* in the Excess Zn + ABA treatment was higher than that in the Basal Zn and Excess Zn treatments. Moreover, in the Excess Zn + AM1 treatment, the expression of most genes except for *VviZIP6* was down-regulated compared with the Basal Zn treatment at 10 DAT.

## Discussion

### ABA Alleviates Physiological Stress on: “Merlot” Grapevines Responding to Excess Zn

Reduced growth of roots and stunted shoot growth are general physiological responses to Zn phytotoxicity in plants (Sresty and Rao, 1999; Hirt and Shinozaki, 2004; Richard et al., 2011). In this study, we show that grapevines exposed for 10 days to toxic concentrations of Zn display reduced root length but show no growth defects or visible toxic symptoms in shoots. This result is in accordance with White et al. (1979) and suggests that shoots are more tolerant to long-term Zn exposure than roots.

Though excess Zn exposure did not affect the growth or the total chlorophyll content in grapevine leaves, it was associated with the inhibition of the net photosynthetic rate as previously reported in poplar (Shi et al., 2015) and beans (Tsonev and Cebola-Lidon, 2012). Given that the Mg^2+^ cofactor can be replaced by Zn^2+^ in chlorophylls (Wettstein et al., 1995), we hypothesize that the Zn-mediated inhibition of photosynthesis in grapevine is due to the impaired functioning of the electron transport within the light harvesting complexes rather than a reduction in the total content of photosynthetic pigments in the antenna.

There is evidence that with excess Zn exposure, higher proportions of the total plant Zn accumulate in the roots, and the excess Zn mainly accumulates in root cortical cell walls or vacuoles (Robson, 1993b; Bringezu et al., 1999). A higher intensity of zinc-dithizone precipitates was consistently observed in root cortical cells, and a larger increase of Zn concentration in roots as compared with other organs analyzed under excess Zn exposure was observed in grapevine seedlings (Supplementary Figure S1 and Figure 1). In the first 4 days of the excess Zn treatments, Zn levels in trunks, stems and leaves generally remained similar to Zn levels in the Basal Zn treatment, indicating that the roots prevented the upper tissues from accumulating potentially toxic Zn levels by accumulating the excess Zn (Figure 1). At 10 DAT, the higher Zn concentrations in roots, trunks, and stems under excess Zn treatments showed the extension of Zn toxicity from the roots to the aerial parts of grapevine seedlings. The addition of exogenous ABA mitigated the accumulation of Zn in roots. This result is consistent with those reported for poplar (Shi et al., 2015). However, in our study the difference between Excess Zn and Excess Zn + ABA was not significant for upper organs.

Plant hormones play important roles in diverse biotic and abiotic stress responses (Bari and Jones, 2009). Although the complex network of interactions of hormones and heavy metal toxicity is not fully understood, evidence has shown that ABA, SA, and JA are involved in the detoxification of heavy metals in plants (Hirt and Shinozaki, 2004; Clemens, 2006; Bari and Jones, 2009; Shi et al., 2015). Interestingly, the response of endogenous ABA concentrations to excess Zn stress varied among studies, including decrease, no change, and increase of ABA in the plant tissues (Zengin, 2006; Yang et al., 2011; Sofo et al., 2013; Shi et al., 2015). In the present study, ABA concentration was not affected by excess Zn, except in the root at 10 DAT, where it was higher in the Excess Zn than in the Basal Zn treatment (Figure 2A,B). On the other hand, exogenous ABA addition consistently increased ABA in roots and leaves, which coincides with previous research (Hsu and Kao, 2003; Shi et al., 2015). As in previous studies that investigated the mitigating effects of ABA on heavy metal toxicity (Hsu and Kao, 2003, 2005; Shi et al., 2015), exogenous ABA was applied simultaneously with excess Zn, and this continuous feeding of the seedlings with ABA (Excess Zn + ABA) produced a high level of ABA in the tissues considered in comparison with the Basal Zn and Excess Zn treatments. The high ABA levels in the leaves might have affected leaf physiology. However, despite a small but significant reduction in leaf photosynthesis observed in the exposed Zn + ABA treatment when compared with the Excess Zn treatment (Table 2), the other leaf parameters assessed were not affected by ABA addition. Transient applications of ABA have also been shown to alleviate lead stress (Zhao et al., 2009; Wang et al., 2013); hence, it would be worth assessing if transient applications would also alleviate Zn toxicity and for how long the alleviating affect would persist in grapevine.

Salicylic acid is also known as one of the key hormones that regulates plant response to biotic and abiotic stress (Maksymiec, 2007; Bari and Jones, 2009), and it plays a key role in the activation of pathogen-induced systemic acquired resistance (SAR) (Kachroo and Robin, 2013). Although there is evidence that an inverse relationship exists between SA-dependent resistance and JA-dependent resistance (Gupta et al., 2000; Turner et al., 2002), the concentration of SA was generally not affected by the treatments while that of JA was (Figure 2C,E). It is worth noticing that ABA treatment decreased SA in roots at 4 DAT compared with the Excess Zn treatment, even though the difference was only marginally significant (*P* = 0.07), indicating that in the grapevine roots exposed to excess Zn, SA signaling could be negatively regulated by ABA as reported for the plant immune response (de Torres Zabala et al., 2009; Kim et al., 2011).

Jasmonic acid plays an important role in plant defense. It is commonly biosynthesized in response to several biotic and abiotic stresses, including toxic action of heavy metals. Many studies have shown that heavy metal stress is closely connected with JA signaling (Xiang and Oliver, 1998; Cobbett, 2000; Maksymiec et al., 2005, Maksymiec, 2007; Bari and Jones, 2009), and it is likely that JA mediates heavy metal-induced gene expression. Additionally, exogenous JA could induce a cross-tolerance to several heavy metal stresses (Xiang and Oliver, 1998; Maksymiec and Krupa, 2006). However, a study of the aquatic plant *Wolffia arrhiza* indicates that exogenous JA acts in a concentration-dependent manner, in which a high concentration of JA enhances heavy metal toxicity (Piotrowska et al., 2009). In this study, the decreased accumulation of JA by excess Zn in roots confirms the role of JA in the adaptation response to heavy metal toxicity (Figure 2E). Furthermore, ABA addition inhibited the decrease of JA concentration in the roots. In addition, it has been reported that JA could protect the photosynthetic apparatus against heavy metal stress (Piotrowska et al., 2009). In our study, Excess Zn + ABA promoted a strong increase of JA concentration in the leaves at 10 DAT; however, this did not mitigate the reduction of photosynthesis determined by excess Zn.

### ABA Induced the Expression Level of Zn-Related Genes in “Merlot” Grapevines Responding to Excess Zn

Zn uptake and transport play pivotal roles in Zn homeostasis in plants, and the Zrt and Irt-like protein (ZIP) family is well characterized for its critical role in Zn uptake, transport, and homeostasis (Astudillo et al., 2013). Our analysis showed that *VviZIP2, VviZIP6, VviZIP7*, and *VviZIP13* homologs are highly expressed in different tissues in grapevine (Supplementary Figure S3). The induced expression of *VviZIP* genes in the Excess Zn treatment supported the fast accumulation of Zn in the roots at 4 DAT. In plants, basic region/leucine zipper motif (bZIP) transcription factors regulate diverse processes including stress signaling (Jakoby et al., 2002) and constitute a group of ABA-response regulators (Hirt and Shinozaki, 2004). *bZIP19* and *bZIP23* were reported to regulate the uptake of Zn (Assunção et al., 2010). The highly induced expression of *VvibZIP23* by ABA addition regardless of treatment time hints at its function as an ABA-response regulator, and it corresponds with the up-regulated expression of ZIP genes in seedlings of the same treatment. The NRAMP family in plants encompasses metal transporters, and transporting ions into the vacuole is one way of reducing toxic metal levels in the cytosol (Hall, 2002). Besides, the transporter NRAMP3 is localized in the vacuolar membrane, and the elevated expression of *NRAMP3* in *Thlaspi caerulescens* was suggested to be necessary for tolerating high Zn concentration (Oomen et al., 2009). In this study, the expression of *VviNRAMP3* was consistently up-regulated in response to the Excess Zn + ABA treatment. However, no induction by excess Zn was found. The detoxification function of *VviNRAMP3* might be induced by ABA alone. There are different mechanisms of tolerance to excess Zn, and there are various proteins involved in these mechanisms (Rout and Das, 2009; Sinclair and Krämer, 2012), which include the chelation of metals in the cytosol (NAS and YSL), the transport of Zn^2+^ from the cytoplasm into vacuoles (HMA), and the pumping of Zn^2+^ out of the cytosol for xylem loading (PCR) (Song et al., 2010; Ueno et al., 2011; Deinlein et al., 2012). The up-regulated expression of these genes by excess Zn in the roots also verified the toxicity of our excess Zn treatments. As for the ABA addition, the stronger and longer-lasting induction of the expression of detoxification genes showed the alleviating effect of exogenous ABA on Zn toxicity in grape seedlings.

Previous research has shown that the activity of antioxidant enzymes highly correlates with Zn levels in plants (Shi et al., 2015), and exogenous ABA causes increased activities of antioxidative enzymes (SOD, APX, CAT, and POD) in plants, relieving the oxidative stress caused by heavy metals (Zhao et al., 2009; Wang et al., 2013). These mechanisms and enzymes might also be involved in the response of grapevines to excess Zn.

### Root Application of AM1 Does Not Alleviate Excess Zn Stress in “Merlot” Grapevines

AM1 was not effective in mitigating Zn uptake. AM1 has been reported as a promising analog of ABA due to its ability to activate ABA receptors. Although, like ABA, AM1 could enhance the tolerance of plants to drought and cold stress, we did find contrasting effects between AM1 and ABA treatments in response to excess Zn in grapevine. Various factors could have led to this inconsistency. First, the exogenous ABA was possibly taken up by the roots and transported into upper organs. On the contrary, seedlings only accumulated AM1 in roots, and there was no evidence of transport of AM1 to other tissues. This possibly contributed to limiting its effects on Zn absorbance and transportation. Second, AM1 is an agonist only for a selective subset of ABA receptors (Cao et al., 2013). Moreover, AM1 is less potent than ABA in binding to certain ABA receptors due to their structural differences. Recently, a series of AM1 fluorine derivatives (AMFs) were verified to be more potent ABA analogs (Cao et al., 2017). Testing the effect of these AMFs on alleviating excess Zn stress in grapevines will provide useful knowledge for their potential application in agriculture.

## Conclusion

Excess Zn exposure led to shorter lateral roots, decreased photosynthesis, fast and high uptake and accumulation of Zn in roots, trunks and stems, decreased concentration of the heavy metal-related endogenous hormone JA, and induced expression of Zn transporter- and detoxification-related genes. Exogenous ABA addition mitigated the uptake and accumulation of Zn and led to higher induced expression of both *VviZIP* genes and detoxification-related genes. These results demonstrate that exogenous ABA could enhance the tolerance of grapevine to excess Zn, likely by regulating the expression of genes involved in Zn uptake and detoxification. As a heavy metal mitigating strategy, treatments with AM1 could not effectively alleviate the accumulation of Zn. Future studies should test if the observed responses are consistent across genotypes and in grafted grapevines.

## Author Contributions

CS and YY performed the experiments. ZZ and SC provided all financial support and critical intellectual input in the design of the study. CS completed the manuscript in collaboration with SC and AR. All authors read and approved the final version of the manuscript.

## Conflict of Interest Statement

The authors declare that the research was conducted in the absence of any commercial or financial relationships that could be construed as a potential conflict of interest.

## Supplementary Material

FIGURE S1 Zn localization in root, trunk, stem, and petiole of “Merlot” (*Vitis vinifera* L.) seedlings exposed to Basal, Excess Zn, and Excess Zn with ABA and AM1 additions by dithizone staining at 10 days after treatment. Well stained samples with zinc-dithizone precipitates (red–purple), arrows point to Zn-dithizone precipitates.

FIGURE S2 ABA mimic 1 (AM1) in the root and leaf tissues of “Merlot” (*Vitis vinifera* L.) seedlings in Excess Zn + AM1 treatment at 4 or 10 days after treatment (*n* = 4). Different letters indicate significantly different averages based on LSD *post hoc* tests.

FIGURE S3 Relative expression of genes (Zrt and Irt-like protein, ZIP; Heavy Metal ATPases of the P1B-type ATPases, HMA; NA Synthase, NAS; Natural Resistance-Associated Macrophage Protein, NRAMP; Yellow Stripe-Like, YSL; Plant Cadmium Resistance, PCR; basic-region leucine zipper transcription factor, bZIP) involved in Zn uptake and transport in different tissues according to whole-genome array data from 14 publicly available experiments. VIT_03s0017g02170, *VviZIP2*; VIT_06s0004g05070, *VviZIP6*; VIT_06s0004g06940, *VviZIP7*; VIT_19s0015g00190, *VviZIP13*; VIT_11s0103g00370, *VviHMA2*; VIT_14s0060g01190, *VviNAS2*; VIT_07s0129g00620, *VviNRAMP3*; VIT_13s0158g00380, *VvibZIP23*; VIT_02s0025g02500, *VviYSL1*; VIT_01s0011g05470, *VviPCR2*.

TABLE S1 Calibration curves for quantification of phytohormones.

TABLE S2 Primer list of tested genes involved in Zn uptake and transport in of “Merlot” (*Vitis vinifera* L.) seedlings.

TABLE S3 Relative expression of 68 grapevine homologous genes across tissues and experimental conditions.
