# Supplementary figures and images for: ABA Alleviates Uptake and Accumulation of Zinc in Grapevine (Vitis vinifera L.) by Inducing Expression of ZIP and Detoxification-Related Genes
Source: Front Plant Sci. 2019 Jul 5;10:872. doi: 10.3389/fpls.2019.00872 (PMC6624748; doi:10.3389/fpls.2019.00872)

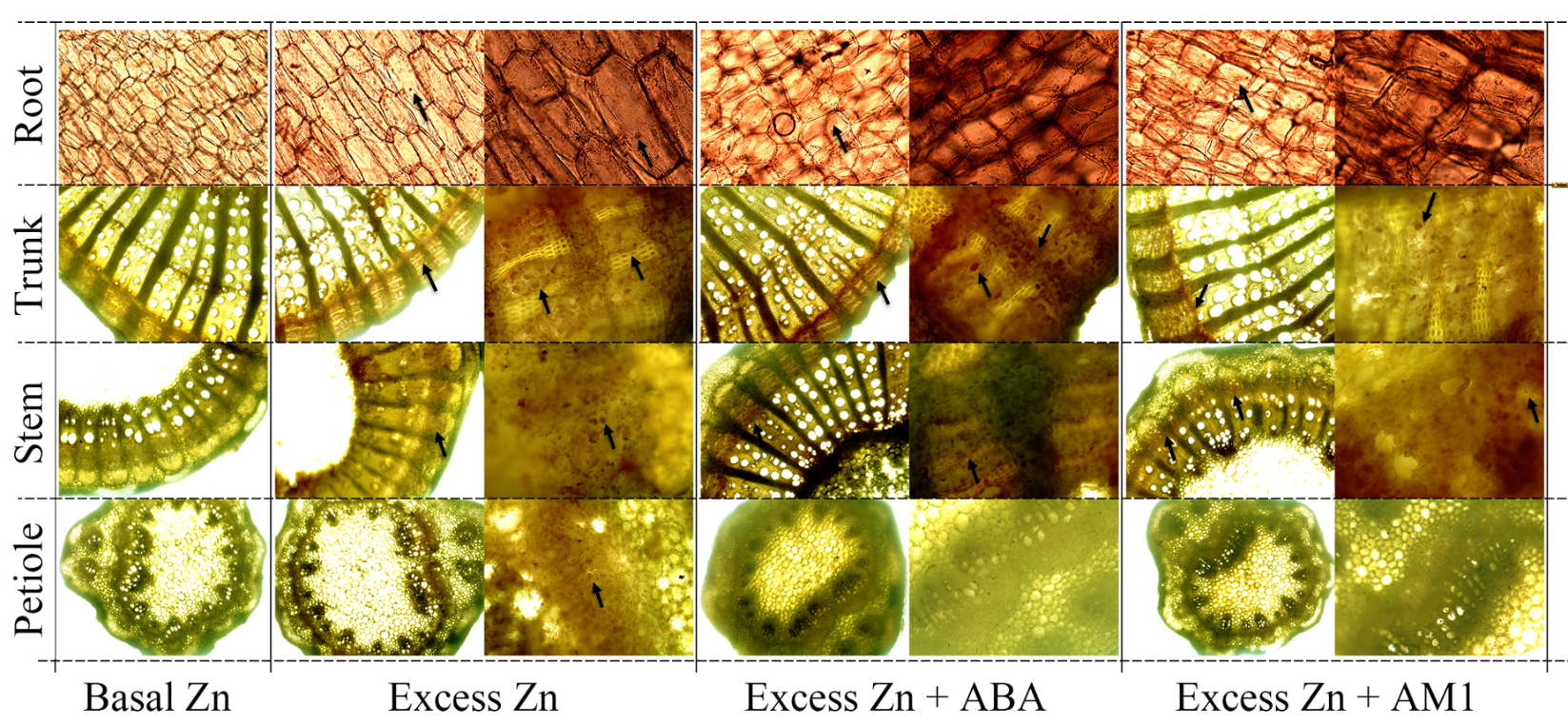

Supplement: FIGURE S1 — Zn localization in root, trunk, stem, and petiole of “Merlot” (Vitis vinifera L.) seedlings exposed to Basal, Excess Zn, and Excess Zn with ABA and AM1 additions by dithizone staining at 10 days after treatment. Well stained samples with zinc-dithizone precipitates (red–purple), arrows point to Zn-dithizone precipitates. [file Image_1.TIF]

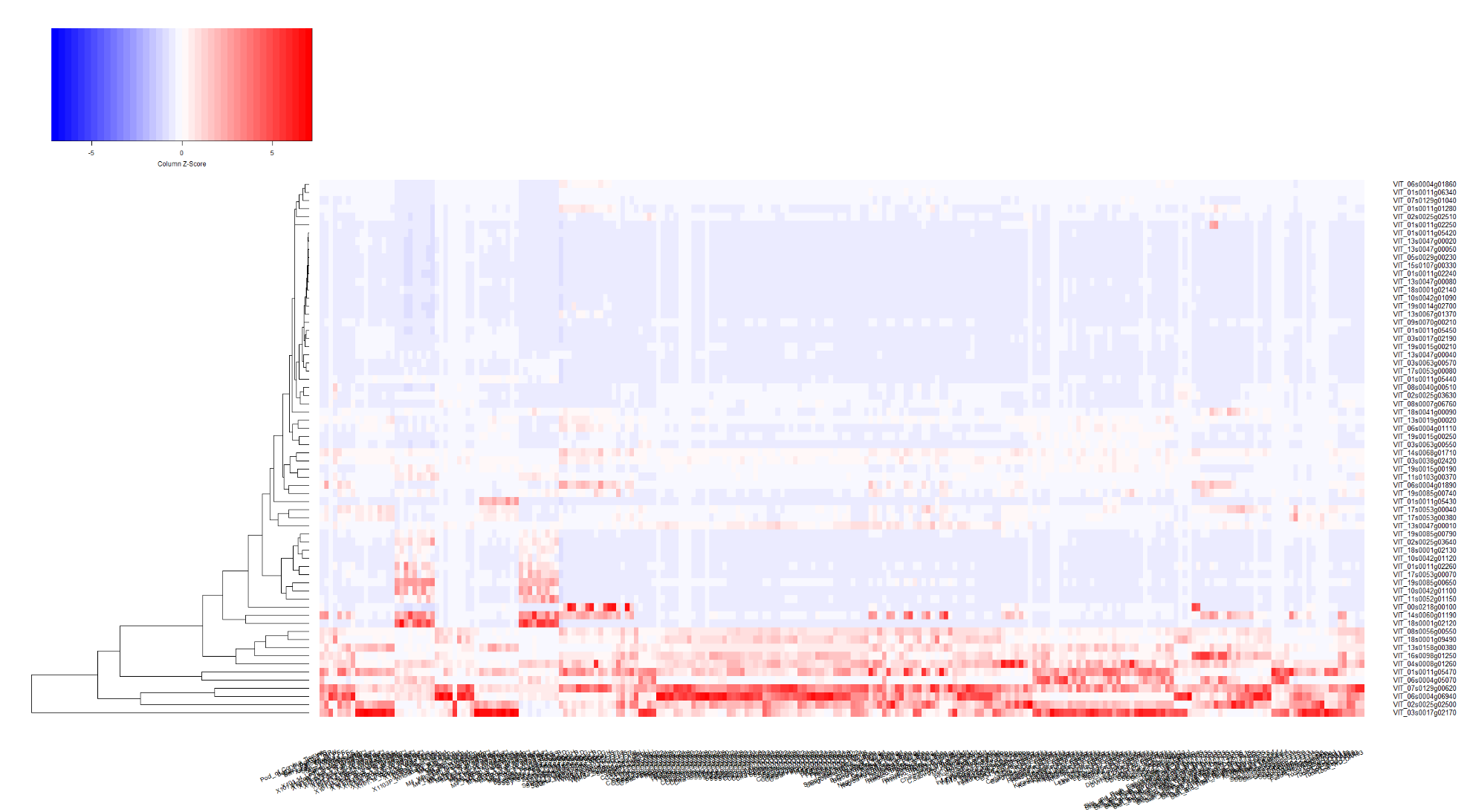

Supplement: FIGURE S3 — Relative expression of genes (Zrt and Irt-like protein, ZIP; Heavy Metal ATPases of the P1B-type ATPases, HMA; NA Synthase, NAS; Natural Resistance-Associated Macrophage Protein, NRAMP; Yellow Stripe-Like, YSL; Plant Cadmium Resistance, PCR; basic-region leucine zipper transcription factor, bZIP) involved in Zn uptake and transport in different tissues according to whole-genome array data from 14 publicly available experiments. VIT_03s0017g02170, VviZIP2; VIT_06s0004g05070, VviZIP6; VIT_06s0004g06940, VviZIP7; VIT_19s0015g00190, VviZIP13; VIT_11s0103g00370, VviHMA2; VIT_14s0060g01190, VviNAS2; VIT_07s0129g00620, VviNRAMP3; VIT_13s0158g00380, VvibZIP23; VIT_02s0025g02500, VviYSL1; VIT_01s0011g05470, VviPCR2. [file Image_3.TIF]
