# Supplementary material for: ABA Alleviates Uptake and Accumulation of Zinc in Grapevine (Vitis vinifera L.) by Inducing Expression of ZIP and Detoxification-Related Genes
Source: Front Plant Sci. 2019 Jul 5;10:872. doi: 10.3389/fpls.2019.00872 (PMC6624748; doi:10.3389/fpls.2019.00872)
Supplement: TABLE S2 — Primer list of tested genes involved in Zn uptake and transport in of “Merlot” (Vitis vinifera L.) seedlings. [file Table_2.docx]

Table S2. Primer list of tested genes involved in Zn uptake and transport in of Merlot (*Vitis Vinifera* cv.) seedlings.

| Gene name | Gene id | Forward primer Sequence (5'->3') | F primer Tm [°C] | Reverse primer Sequence (5'->3') | R primer Tm [°C] | Length |
| --- | --- | --- | --- | --- | --- | --- |
| *VviZIP2* | VIT_03s0017g02170 | ATGAACCCAAGGATGCAGGG | 60 | CATTTGGCCAGGAGGGACAT | 60 | 95 |
| *VviZIP6* | VIT_06s0004g05070 | TTTCTTGCTGTGCTTGCTGG | 60 | TCACAACAAACAAGGATCGTTTACA | 60 | 149 |
| *VviZIP7* | VIT_06s0004g06940 | CCTGACAAACAAGGTGAAGA | 60 | CTATGATGACCGAGTGGAATATG | 60 | 94 |
| *VviZIP13* | VIT_19s0015g00190 | CGGACAGCGTTGACTTGA | 60 | CTGCGTTCCCAGCACTAG | 60 | 143 |
| *VviHMA2* | VIT_11s0103g00370 | GGAAGGACCAACAGCCATGA | 60 | AACCACATGCCCTGTCTCTG | 60 | 124 |
| *VviNAS2* | VIT_14s0060g01190 | GGGCTGTCAGGATGAGCTTT | 60 | GCGGACTAGTTGGGTGAACA | 60 | 118 |
| *VviNRAMP3* | VIT_07s0129g00620 | GGTACGGAACAAGCCAATAA | 60 | CAGCTGCTAGTAACCCAATAC | 60 | 118 |
| *VvibZIP23* | VIT_13s0158g00380 | TAGTATTAGTGGGAGTGGGAAA | 60 | GTCCTTCAGCACTTACCATATC | 60 | 107 |
| *VviYSL1* | VIT_02s0025g02500 | AGGTGATGGACTCTACAATTTC | 60 | GTCTGCTCATCTCCATCTATTT | 60 | 111 |
| *VviPCR2* | VIT_01s0011g05470 | ACTGCTCTCTTGATTTCCTTT | 60 | CATGGTGGATCGGAAGAATTA | 60 | 79 |
